# Breast Cancer Treatment Delay in SafetyNet Health Systems, Houston Versus Southeast Brazil

**DOI:** 10.1093/oncolo/oyac050

**Published:** 2022-03-28

**Authors:** Maryam Nemati Shafaee, Leonardo Roberto Silva, Susana Ramalho, Maira Teixeira Doria, Rodrigo De Andrade Natal, Victor Cabello, Livia Cons, Marina Pavanello, Luiz Carlos Zeferino, Max S Mano, Rudinei Diogo Marques Linck, Leticia Souza Batista, Estela Pantarotto Pedro, Bruno Henrique De Paula, Gustavo Zuca-Matthes, Emily Podany, Shalini Makawita, Kelsey Ann Stewart, Spiridon Tsavachidis, Rull Tamimi, Melissa Bondy, Logan Debord, Matthew Ellis, Jose Bines, Cesar Cabello

**Affiliations:** 1 Baylor College of Medicine, Houston, TX, USA; 2 Department of Obstetrics and Gynecology, Faculty of Medical Sciences, State University of Campinas (UNICAMP), Campinas, São Paulo, Brazil; 3 Department of Obstetrics and Gynecology, Clinical Hospital of Federal University of Paraná, Curitiba, Paraná, Brazil; 4 School of Women’s and Children’s Health, Lowy Cancer Research Centre, University of New South Wales, Sydney, Australia; 5 Hospital Sírio-Libanês, São Paulo, Brazil; 6 Instituto Nacional Do Câncer (INCA – HCIII), Rio de Janeiro, Brazil; 7 Barretos Cancer Hospital, Barretos, Brazil; 8 MD Anderson Cancer Center, Houston, TX, USA; 9 Department of Obstetrics and Gynecology, University of Minnesota, Minneapolis, MN, USA; 10 Department of Population Health Sciences, Weill Cornell Medicine, New York-Presbyterian, New York, NY, USA; 11 Center for Population Health Sciences, Stanford Cancer Institute, Stanford, CA, USA; 12 Department of Dermatology, Anschutz Medical Campus, University of Colorado, Aurora, CO, USA

**Keywords:** breast cancer, treatment delay, patient delay, outcomes

## Abstract

**Background:**

Breast cancer outcomes among patients who use safety-net hospitals in the highly populated Harris County, Texas and Southeast Brazil are poor. It is unknown whether treatment delay contributes to these outcomes.

**Methods:**

We conducted a retrospective cohort analysis of patients with non-metastatic breast cancer diagnosed between January 1, 2009 and December 31, 2011 at Harris Health Texas and Unicamp’s Women’s Hospital, Barretos Hospital, and Brazilian National Institute of Cancer, Brazil. We used Cox proportional hazards regression to evaluate association of time to treatment and risk of recurrence (ROR) or death.

**Results:**

One thousand one hundred ninety-one patients were included. Women in Brazil were more frequently diagnosed with stage III disease (32.3% vs. 21.1% Texas; *P* = .002). Majority of patients in both populations had symptom-detected disease (63% in Brazil vs. 59% in Texas). Recurrence within 5 years from diagnosis was similar 21% versus 23%. Median time from diagnosis to first treatment defined as either systemic therapy (chemotherapy or endocrine therapy) or surgery, were comparable, 9.9 weeks versus 9.4 weeks. Treatment delay was not associated with increased ROR or death. Higher stage at diagnosis was associated with both increased ROR and death.

**Conclusion:**

Time from symptoms to treatment was considerably long in both populations. Treatment delay did not affect outcomes.

**Impact:**

Access to timely screening and diagnosis of breast cancer are priorities in these populations.

## Teaser

This article examines the impact of treatment delay on the outcomes of patients with breast cancer, comparing results of those utilizing the public health systems in Harris County (Texas, USA) and Southeast Brazil.

**Implications for Practice**

Patients with breast cancer who utilize the Harris Health hospitals in Houston, Texas have similar outcomes to patients with breast cancer who use public hospitals in Southeast Brazil. Long patient delay, issues of access, and early diagnosis are pertinent to both populations. The authors’ findings may help dispel the conviction that the good outcomes for breast cancer reported in the United States may be a function of receiving cancer care in a high-income country. On the contrary, these findings suggest that patients of low socioeconomic status who cannot afford commercial insurance and therefore utilize public safety-net hospitals in the United States could have similar breast cancer outcomes as patients who receive care across public hospitals in the middle-income country of Brazil. These findings highlight the importance of policies that prioritize access to timely screening and diagnosis of breast cancer.

## Introduction

Breast cancer is the most common cancer among women worldwide with the yearly incidence rate of nearly 2 million.^1^ Incidence and survival rates of breast cancer vary across the world.^2^ Many factors underlie the global disparities in outcomes of breast cancer, including genetics, access to screening and treatment, as well as population structure.^3^ Over the past 50 years, the yearly incidence of breast cancer has increased in the United States, however, survival from breast cancer has improved.^4,5^ In developing countries incidence rates of breast cancer are increasing, which has been partly attributed to the implementation of mammography screenings as well as increase in public awareness.^1,6^ Low- and middle-income countries (LMICs) have lower incidence of breast cancer than high-income countries (HICs), but mortality rates in the majority of these countries are higher.^7^ Only 5% of the total global cancer care resources are spent in LMICs,^8^ where cost-effective investments in prevention, early diagnosis, and access to care are priorities.^9^ Global annual breast cancer deaths are projected to near 1 million by 2030, and over 70% of these deaths would be occurring in LMICs.^10^

In Brazil, the 5-year overall survival of breast cancer was 75.25% in 2018.^11^ This figure is much lower than the survival rates of invasive breast cancer in HICs such as the United States (90%), Japan (89%), and Norway (98%).^10,11^ Social inequality is a severe issue in Brazil, not only in terms of income, but also related to race, sex, and access to employment, housing, and health care. Brazil’s southeast region is an area of interest for studying health outcomes as the region has long been the main destination for people migrating from rural areas, leading to increased urbanization and breast cancer rates. The increase in life expectancy and lower parity rates also contributed to a higher breast cancer incidence in this region, mirroring the rates in HICs.^12^

While many states within the United States have experienced a decline in breast cancer mortality, Texas is among a handful of states with a recent increase in breast cancer mortality rates.^4,13^ Harris County of Texas, the third-most populous county in the United States and among the fastest-growing, has some of the worst outcomes of breast cancer in the country.^14-16^ Racial minorities, including Hispanic and African American residents, are growing populations in Harris County, making up over 60% of this county’s population.^17^ Outcomes for variety of health-related indicators are poor among minority populations in this county, where lower access to preventive services and poverty are growing concerns. In Harris County, 27% of mammogram eligible women, ages 40-64 are uninsured versus 11% national average.^18^

Early diagnosis improves breast cancer outcomes. Mammogram screening is consistently associated with breast cancer mortality reduction.^19-22^ Advanced stage at diagnosis, high tumor grade, and absence of hormone receptor positivity adversely affect prognosis.^23-25^ Delay in diagnosis or treatment initiation is a modifiable determinant of outcomes. Delays can be classified as (1) patient delay, the period between the onset of symptoms and first medical consultation; and (2) system delay, time from the first consultation to definitive diagnosis, “diagnosis delay,” or treatment initiation, “treatment delay”.^26,27^ Patient delay of longer than 3 months is a risk factor for advanced stage at diagnosis.^26,28-33^ For metastatic disease, treatment delay of 12 weeks or more is associated with worse survival.^34^ In nonmetastatic disease, the negative impact of >12 weeks treatment delay seems to be conferred through more advanced stage at time of treatment initiation.^35^ A retrospective analysis of Surveillance, Epidemiology, and End Results (SEER) Medicare cohort reported incremental reductions in survival with every 30-day delay from diagnosis to surgery (hazard ratio [HR], 1.09; 95% CI, 1.06-1.13; *P* < .001).^36^ An MD Anderson retrospective review suggested that 8 weeks or longer delay from diagnosis to neoadjuvant chemotherapy maybe be associated with higher risk of death (HR 1.19; 95% CI, 0.99-1.44).^37^ Another MD Anderson study reported delay of 91 days or longer from surgery to systemic chemotherapy to be associated with increased risk of death (HR 1.34; 95% CI, 1.15-1.57).^38^ Delay in initiation of adjuvant and/or neoadjuvant chemotherapy seems to particularly affect outcomes in high-risk subtypes (eg, triple-negative breast cancer [TNBC] and Her2 positive disease).^38-40^ Some studies have found no association between treatment delay and risk of death from breast cancer.^41,42^ Longer time to treatment (TTT) has been associated with better prognosis in two studies.^43,44^ The reasons for such unexpected findings are assumed to be providers’ practice patterns that prioritize urgent treatment of aggressive disease.^25^ The effect of treatment delay on different stages and subtypes of breast cancer were not clearly compared in these studies.

While studies on delay in developing countries have generally focused on patient delay, system delay is more frequently studied in developed countries.^45^ Direct comparisons of delay parameters between developed and developing countries in breast cancer are few.^46,47^ Such studies may uncover delay determinants which can, in turn, serve as indirect measures of health systems quality.^47^

In this study, as our primary aim, we compared treatment delay, defined as time from diagnosis to first treatment, and its potential adverse effect on breast cancer outcomes among patients utilizing public services in southeast Brazil and Harris Health in Harris County Texas. The population served by these health services is often unable to afford commercial insurance and is generally of lower socioeconomic status (SES). They experience worse outcomes of breast cancer compared to their national averages.^48,49^ One study at Barretos Cancer Hospital in southeast Brazil reported longer duration of symptoms among breast cancer patients 9.5 months versus 6.5 months in the SEER Medicare population.^47^ At Harris health in 2010, median time from first abnormal mammogram to pathologic diagnosis was 89 days, and median time from diagnosis to treatment was 121 days.^50^

## Methods

We performed a retrospective chart review of patients with breast cancer at three public hospitals in southeast Brazil; Women’s Hospital Pinotti, Barretos Cancer Hospital, and Brazilian National Institute of Cancer Hospital, and two Harris Health safety net hospitals in Harris County; Ben Taub Hospital and London B. Johnson Hospital. The study was approved by the Institutional Review Board at all participating institutions. Patients with stage I to III invasive primary breast cancer who received their first treatment between January 1, 2009, and December 31, 2011, were included in the study. Patients with noninvasive disease, de novo metastatic disease, or those no follow-up visits were excluded.

The following information was abstracted from patients’ charts; age at diagnosis, ethnicity, menopausal status, family/personal history of cancer, date of patient-reported symptoms onset, date of first abnormal radiological examination, date of the first visit at the institution, and date of histological diagnosis. Tumor-related data recorded included histological type, histological grade, estrogen receptor (ER), progesterone receptor (PgR), human epidermal growth factor receptor 2 (HER2), and Ki67 status. We defined breast cancer subtype as luminal (ER and/or PgR positive and HER2-negative), HER2-positive (HER2-positive regardless of the ER/PgR status), and TNBC (ER/PgR/HER2-negative). Clinical stage at diagnosis and pathological staging at surgery were recorded according to the American Joint Committee on Cancer/International Union Against Cancer Tumor, Node, Metastasis staging 7th edition.

The first therapy received by the patient was defined as surgery or systemic therapy (neoadjuvant chemotherapy/neoadjuvant endocrine therapy). Chemotherapy was classified as anthracycline-based, anthracycline and taxane-based, or other, and endocrine therapy as an aromatase inhibitor and/or tamoxifen. For patients with HER2-positive disease, history of trastuzumab therapy was recorded.

### Statistical Analysis

Categorical variables were compared between the Harris Health population and that of Southern Brazil by Fisher exact test. Continuous variables were compared by nonparametric Mann–Whitney test. Time of diagnosis was defined as the date of histologic diagnosis, and in the absence of that information, time at first visit at the cancer clinic was taken as the time of diagnosis. TTT was defined as the time (in weeks) from diagnosis to the first therapy (surgery or systemic therapy). Time to first systemic therapy (TFST) was defined as the time (in weeks) from diagnosis to the first systemic therapy (neoadjuvant or adjuvant). Time intervals were categorized in keeping with published studies from different health systems on optimizing TTT^34,51,52^ where national average for TTT at large academic centers is approximately 6 weeks^53^ and delays of 8 weeks and 12 weeks further increase the risk of death.^54^ Five categories for TTT interval were considered which included < 6 weeks, 6-8 weeks, 8-12 weeks, 12-24 weeks, and > 24 weeks where < 6 weeks was taken as baseline for comparison. TTT, TFST, and time from symptoms to first therapy were then compared between the two populations. We defined poor outcomes as breast cancer recurrence or death from breast cancer within 5 years from diagnosis. Disease-free survival was measured from the date of diagnosis to the date of first documented recurrence (local or distant). Overall survival was defined as the time from diagnosis to date of death within a 5-year follow-up period. Risk of recurrence (ROR) or death was analyzed using Cox proportional hazards regression to estimate the HR associated with every 30-day delay in treatment and recurrence or death. Two separate multivariate regression analyses were performed evaluating the effect of TTT and TFST on ROR or death. The analysis was adjusted for potential confounders including age, breast cancer subtypes, and stage at diagnosis. Patients without recurrence were censored at the date of the last follow-up visit. Those without at least 24 weeks of follow-up data were excluded from survival analysis.

All statistical tests were two-sided. *P* values ≤ .05 were considered statistically significant. The data were collected and managed using Redcap (hosted by the State University of Campinas), and statistical analysis was carried out by using SAS 9.4.

## Results

A total of 1191 cases (Southeast Brazil, *n* = 963; Harris County, Texas, USA, *n* = 228) were identified for planned data abstraction. Date of diagnosis was missing for 181/1191 (15.2%) patients for whom date of first cancer clinic visit was taken as proxy for date of diagnosis. Date of symptoms development was missing for 524/1191 (44%) patients. Seven patients with less than 24 weeks of follow-up were excluded. Follow-up time was censored at 5 years from diagnosis.

Table 1 lists the patients’ characteristics. Women in Southeast Brazil were older at diagnosis (55.7 years vs. 53.1 years; *P* = .002), less frequently diagnosed with stage I (31.0% vs. 39.9%; *P* = .002) and more often presented with stage III disease (32.3% vs. 21.1%; *P* = .002). Subtype distribution was similar, however, women in Southeast Brazil received neoadjuvant chemotherapy less frequently than the Harris County patients (23.2% vs. 37.9%; *P* < .001). The racial categories of the two populations were not directly comparable as the race in the Brazilian population was not self-defined. Majority of the patients in both populations presented with symptom-detected breast cancer, 59% in Harris County and 63% in Southeast Brazil. There were no differences in terms of recurrence and 5-year overall survival between the two populations (Table 1).

**Table 1.** Patient characteristics by demographic data, clinical/tumor factors, treatment, and long-term outcomes according to the place of diagnosis.

Characteristics	Harris County, Texas, USA (*n* = 228)	Southeast Brazil (*n* = 963)	*P*
Age (median, years)	53.1	55.7	.002
Race			<.001
White	28 (12.2)	632 (83.3)	
African American	78 (33.9)	36 (4.7)	
Asian	14 (6.1)	4 (0.5)	
Native American	1 (0.4)	1 (0.1)	
Mixed races	0 (0)	84 (11.1)	
US only Hispanic	109 (47.4)	2 (0.3)	
Menopausal status			.096
Premenopausal	74 (32.7)	317 (33.1)	
Post-menopausal	134 (59.3)	598 (62.5)	
Unknown	18 (8.0)	42 (4.4)	
Method of detection			.314
Symptomatic	130 (59.1)	578 (63.0)	
Screening	90 (40.9)	340 (37.0)	
Stage			.002
I	91 (39.9)	299 (31.0)	
II	89 (39.0)	353 (36.7)	
III	48 (21.1)	311 (32.3)	
Subtype			.738
Luminal	143 (63.3)	574 (62.4)	
HER2-positive	44 (19.5)	199 (21.6)	
Triple-negative	39 (17.3)	147 (16.0)	
Neoadjuvant chemotherapy			<.001
No	144 (62.1)	742 (76.8)	
Yes	88 (37.9)	224 (23.2)	
Recurrence within 5 years	34 (23.0)	152 (21.0)	.66
Death within 5 years	14 (10.0)	96 (14.0)	.27

Note: Data are presented as No (%), unless otherwise indicated.

Abbreviations: HER2, human epidermal growth factor receptor 2.

Table 2 shows how many subjects fell into each delay interval category. Less than 20% of patients from both locations had a TTT of less than 6 weeks. Approximately 10% of patients from Harris County and Southeast Brazil had an interval of less than 6 weeks for TFST. Comparing TTT and TFST between the two populations, TTT was not found to be significantly different, however, TFST was significantly longer in Southeast Brazil (median 19.5 weeks vs. 16.1 weeks; *P* < .001). Time from onset of symptoms to treatment was not significantly different, while time from symptoms to first systemic therapy was longer in patients from Southeast Brazil (median 41.7 weeks vs. 35.5 weeks; *P* = 0.027) (Table 3).

**Table 2.** Delay intervals by place of diagnosis.

	Harris County, Texas, USA (*n* = 228)	Southeast Brazil (*n* = 963)
Time to first treatment		
<6 weeks	41 (18.8)	130 (15.7)
6-8 weeks	37 (17)	119 (14.3)
8-12 weeks	58 (26.6)	293 (35.3)
12-24 weeks	66 (30.3)	210 (25.3)
>24 weeks	16 (7.3)	78 (9.4)
Time to first systemic treatment		
<6 weeks	24 (11.8)	72 (8.8)
6-8 weeks	19 (9.4)	29 (3.5)
8-12 weeks	31 (15.3)	76 (9.3)
12-24 weeks	83 (40.9)	376 (46)
>24 weeks	46 (22.7)	265 (32.4)

**Table 3.** Time to the start of treatment in weeks, by place of diagnosis.

	Harris County, Texas, USA (*n* = 228)	Southeast Brazil (*n* = 963)	*P*
Time from diagnosis to first treatment, mean (median)	14.3 (9.4)	12.4 (9.9)	.672^a^
Time from diagnosis to first systemic therapy, mean (median)	20.9 (16.1)	21.7 (19.5)	<.001^a^
Time from onset of symptoms to first treatment, mean (median)	43.4 (32.8)	45.9 (34.3)	.065^a^
Time from onset of symptoms to first systemic therapy, mean (median)	49.0 (35.5)	52.1 (41.7)	.027^a^

Mann-Whitney.

Women in Southeast Brazil experienced much shorter TTT in days when presenting with triple-negative (mean: 74.7 days; *P* = .003), HER2-positive (mean: 76.1 days; *P* = .005), or stage III breast cancer (mean: 78.9 days; *P* = .03) compared to stage I or stage II luminal breast cancer (93.0 days) (Table 4). Women treated in Harris County, Texas, USA had shorter TFST if diagnosed with stage III disease (92.0 days; *P* = .009) compared to stages I or II luminal breast cancer (164.4 days) (Table 4). The results of multivariate regression analysis were significant for increased ROR associated with stage III (HR 6.74; 95% CI, 2.41-18.83; *P* < .001) and TNBC (HR 3.84; 95% CI, 1.84-7.99; *P* < .001) among the women treated at Harris County, Texas, USA.

**Table 4.** Time to treatment according to stage and subtype.

Time to first treatment (in days)								
Groups	Harris County, Texas, USA				Southeast Brazil			
	*N*	Mean time to first treatment (IQR)	*t*-statistic	*P*	*N*	Mean time to first treatment (IQR)	*t*-statistic	*P*
Stage I or II/ER+/PgR+	116	102.7 (53.0)	REF		435	93.0 (42.0)	REF	
TNBC	39	69.6 (18.0)	1.6	.112	147	74.7 (48.5)	2.96	.003
HER2+	44	180.9 (45.5)	−1.44	.157	199	76.1 (40.5)	2.8	.005
Stage III	48	126.8 (65.0)	−0.45	.651	311	78.9 (46.0)	2.16	.030

Time to first systemic treatment (in days)								
Groups	Harris County, Texas, USA				Southeast Brazil			
	*N*	Mean time to first systemic therapy (IQR)	*t*-statistic	*P*	N	Mean time to first systemic therapy (IQR)	*t*-statistic	*P*
Stage I or II/ER+/PgR+	116	164.4 (87.0)	REF		435	171.6 (76.0)	REF	
TNBC	39	154.9 (66.0)	0.14	.886	147	121.3 (99.0)	5.81	<.001
HER2+	44	124.1 (88.7)	1.26	.210	199	129.2 (100.2)	5.18	<.001
Stage III	48	92.0 (77.0)	2.63	.009	311	103.4 (101.5)	8.52	<.001

Abbreviations: ER, estrogen receptor; HER2, human epidermal growth factor receptor 2; IQR, interquartile range; PgR, progesterone receptor; TNBC, triple-negative breast cancer.

Regression analyses for TTT according to 30-day delay increments are shown in Table 5. The HRs were calculated using as reference stage I and luminal subtype. In Southeast Brazil, factors associated with increased ROR were stage II (HR 2.77; 95% CI, 1.62-4.75; *P* < .001) and stage III disease (HR 6.25; 95% CI, 3.72-10.49; *P* < .001). In Harris County, stage III (HR 6.64; 95% CI, 2.44-18.13; *P* < .001) and TNBC (HR 3.99; 95% CI, 1.95-8.18; *P* < .001) were associated with increased ROR. Similar results were observed regarding TFST. Predictors of higher rate of recurrence were stage II (HR 3.15; 95% CI, 1.77-5.61; *P* < .001) and stage III (HR 6.81; 95% CI, 3.85-12.07; *P* < .001). For Harris County patients, stage III (HR 5.76; 95% CI, 2.09-15.90; *P* < .001) and TNBC (HR 4.21; 95% CI, 2.06-8.60; *P* < .001) were independent predictors of recurrence (Table 6). Kaplan-Meier curves for overall survival according to TTT for Harris County and Southeast Brazil are shown in Supplementary Figure 1.

**Table 5.** Cox proportional hazards of time to first treatment.

	HR^a^ (95% CI)	*P*
Harris County, Texas, USA		
Time to first treatment (months)	0.96 (0.84-1.10)	.57
Stage		
I	1 (ref)	
II	2.56 (0.92-7.12)	.07
III	6.64 (2.44-18.13)	<.001
Subtype		
Luminal	1 (ref)	
HER2+	1.14 (0.44-2.95)	.78
TNBC	3.99 (1.95-8.18)	<.001
Southeast Brazil		
Time to first treatment (months)	0.93 (0.86-1.01)	.087
Stage		
I	1 (ref)	
II	2.77 (1.62-4.75)	<.001
III	6.25 (3.72-10.49)	<.001
Subtype		
Luminal	1 (ref)	
HER2+	1.13 (0.78-1.64)	.51
TNBC	1.05 (0.70-1.59)	.79

HR represents the increase in risk associated for an added 30-day of time to first treatment delay.

Abbreviations: HR, hazard ratio; ref, reference; TNBC, triple-negative breast cancer.

**Table 6.** Cox proportional hazards of time to first systemic treatment.

	HR^a^ (95% CI)	*P*
Harris County, Texas, USA		
Time to first systemic treatment (months)	0.97 (0.88-1.07)	.51
Stage		
I	1 (ref)	
II	2.09 (0.74-5.86)	.16
III	5.76 (2.09-15.90)	<.001
Subtype		
Luminal	1 (ref)	
HER2+	0.90 (0.33-2.51)	.85
TNBC	4.21 (2.06-8.60)	<.001
Southeast Brazil		
Time to first systemic treatment (months)	0.96 (0.90-1.01)	.14
Stage		
I	1 (ref)	
II	3.15 (1.77-5.61)	<.001
III	6.81 (3.85-12-07)	<.001
Subtype		
Luminal	1 (ref)	
HER2+	1.15 (0.79-1.67)	0.45
TNBC	1.03 (0.68-1.56)	0.88

HR represents the increase in risk associated for an added 30-day of time to first systemic treatment delay.

Abbreviations: HR, hazard ratio; ref, reference; TNBC, triple-negative breast cancer.

## Discussion

Brazil is a middle-income country with a tax-funded public health system called Sistema Único de Saúde (SUS) with universal coverage. SUS provides care to >58% of the population but suffers from limited resources, and regional variations in coverage may lead to delays in diagnosis.^55^ In 2003, almost 50% of the women aged 50 and older covered under SUS never had a screening mammogram.^12^ Less than 25% of the population in Brazil can afford private insurance.^56^

Harris Health is a state-funded program that provides screening as well as comprehensive cancer care to the population residing within the county. Over 60% of the population that receives care at Harris Health has an annual income of less than $25K, and the majority are unable to afford commercial insurance. The clinics accept patients on Medicare (18%), certain commercial insurance holders (13%), as well as affordable care act recipients.

In our study, we found that women in southeast Brazil were more frequently diagnosed with stage III disease and generally received less neoadjuvant therapy compared to the Harris health population. Both populations, however, had similar recurrence and death rates within the 5-year follow-up. Median TFST was longer in Southeast Brazil, even for symptomatic patients, when compared to Harris County. In Southeast Brazil, women presenting with high-risk disease (HER2-positive, TNBC, stage III) were treated earlier compared to lower-risk women (luminal stage I-II disease). Similarly, in Harris health, TTT was shorter for patients diagnosed with stage III disease compared to those who were diagnosed with stage I-II luminal breast cancer. The multivariate analysis was significant for higher rates of recurrence or death for TNBC, as well as stages II-III. Delays in TTT and TFST were not associated with higher ROR in either population.

Previous studies have reported that in Brazil, a substantial number of women are diagnosed with locally advanced disease due to poor awareness and lack of access to screening programs or treatment centers.^57,58^ In our study, we found that one-third of Brazilian women were diagnosed with stage III disease, which is consistent with Brazilian National Cancer Institute data and somewhat higher than what was shown by the AMAZONA study.^58^

Our analyses did not show differences in terms of subtype distribution among Brazilian and Harris County women, despite imbalances in ethnic background.^59^ Although the population in Brazil had a higher proportion of stage III disease for which neoadjuvant therapy is standard, this strategy was less frequently adopted when compared to Harris County women. In fact, Simon and cols found that neoadjuvant therapy was administered to only 18.8% of Brazilian women while a higher proportion would qualify for this approach.^58^

It is worth noting that while both populations in this study have limited access to care, the overall five-year recurrence and survival rate did not differ from other published data for Brazil, other South American countries, and the United States.^11,58,60^ A potential explanation for this observation may be that after patients have already established care and begun treatment, the outcomes adjust towards the regional baseline. In other words, the disparity is a more pressing issue at the point of access to care rather than quality of care and services rendered thereafter. Of particular interest are the reports that some cities in southeast Brazil have shown a reduction in mortality due to improvements in access to screening services.^57,60^

The two populations in this study were not directly comparable in terms of racial categories given that the race was self-defined in the Harris Health population but designated by the clinic staff in Brazil. However, the breakdown of symptom versus screen-detected breast cancer was similar in the two populations emphasizing that socioeconomic disparities and limited access to care and screening services affect both populations.^61^

We found no difference in TTT between women treated at Harris County and Southeast Brazil, but TFST was longer for Brazilian women. This can be explained by the higher percentage of women in Harris County that received neoadjuvant therapy. Our analysis of TTT in Brazilian patients is in accordance with a prior report in a public hospital in Southeast Brazil, where on average it takes about 8 weeks for women to start treatment for breast cancer.^62^ Nevertheless, most of the delays were related to the time it took for the patient to get the first consultation, similar to the report by Redondo et al^31^ in which patient delay, defined as the interval from the start of symptoms to the first hospital visit, was significantly longer than diagnosis and treatment delay.

TTT longer than 12 weeks was associated with a lower ROR in Southeast Brazil. Although it would seem a paradox, we note that patients with higher stage disease and more aggressive pathology (TNBC and Her2 positive) or higher stage have shorter TTT compared to those with luminal pathology. In Harris County, delays in TTT and TFST were not associated with higher ROR, regardless of stage and subtype. These data on TFST is possibly confounded by differences in treatment sequencing, as most patients with stage III disease received neoadjuvant therapy. On the other hand, patients with luminal breast cancer had surgery as their first treatment modality. One may consider that women with more aggressive disease and advanced stage are prioritized to initiate treatment more urgently, usually with neoadjuvant systemic therapy. It is imperative to note that our follow-up for patients with luminal tumors is relatively short as these cancers often recur later than the other subtypes. Therefore, either delay did not impact survival or our analysis was not sufficiently powered to detect its effect.

The retrospective nature of our study is a potential limitation. We could not accurately verify the date of first symptoms for most patients, as this information was not often registered in medical records. Also, we did not retrieve data on sociodemographic and economic factors, which may affect delay outcomes. We acknowledge that our results may not reflect national figures for both countries due to significant regional social and economic inequalities. However, the participating institutions are regional high-volume reference centers for breast cancer care, with proper patient follow-up.

In conclusion, treatment delay was not found to be associated with higher risk of recurrent disease or death in either of the two cohorts of low SES patients in Texas and Southeast Brazil. Future studies are needed to further assess the impact of delay per breast cancer subtypes, so that in resource-limited settings, provider practices can be appropriately modified to improve outcomes in a risk-stratified manner.

## Supplementary Material

oyac050_suppl_Supplementary_Figures

## Data Availability

The data underlying this article will be shared on reasonable request to the corresponding author.
