# Supplementary material for: Breast Cancer Treatment Delay in SafetyNet Health Systems, Houston Versus Southeast Brazil
Source: Oncologist. 2022 Mar 28;27(5):344–51. doi: 10.1093/oncolo/oyac050 (PMC9074991; doi:10.1093/oncolo/oyac050)

Figure 1. Kaplan-Meier estimates of overall survival according to time to first treatment

A. Harris County, Texas/USA


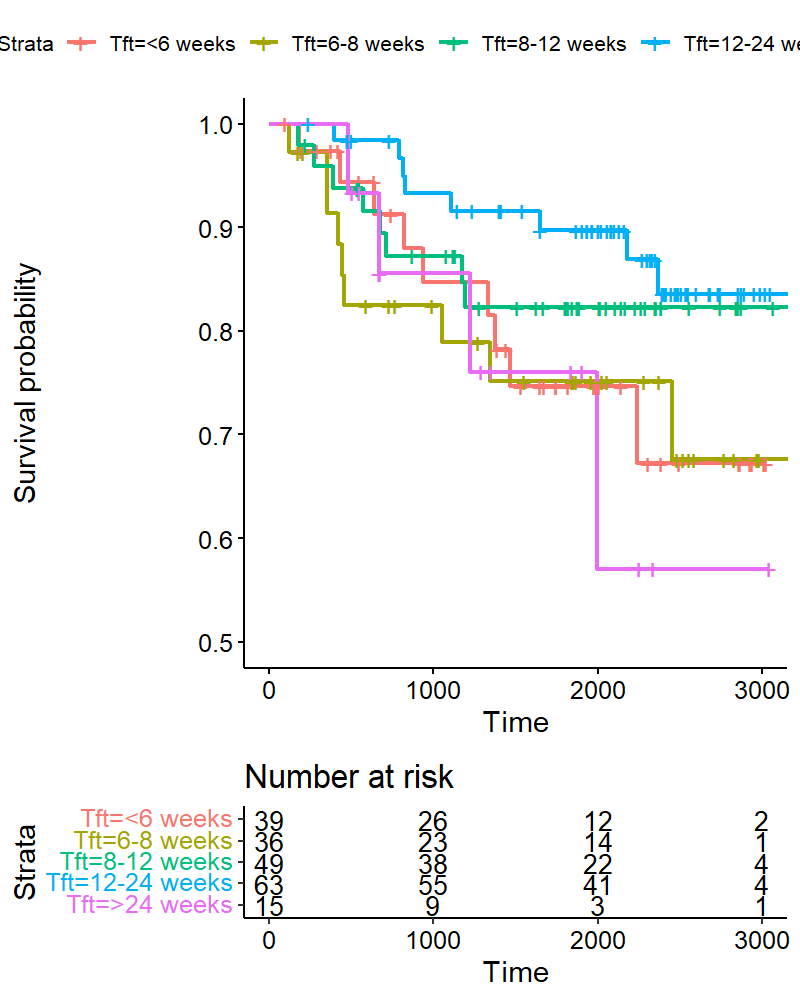


B. Southeast Brazil


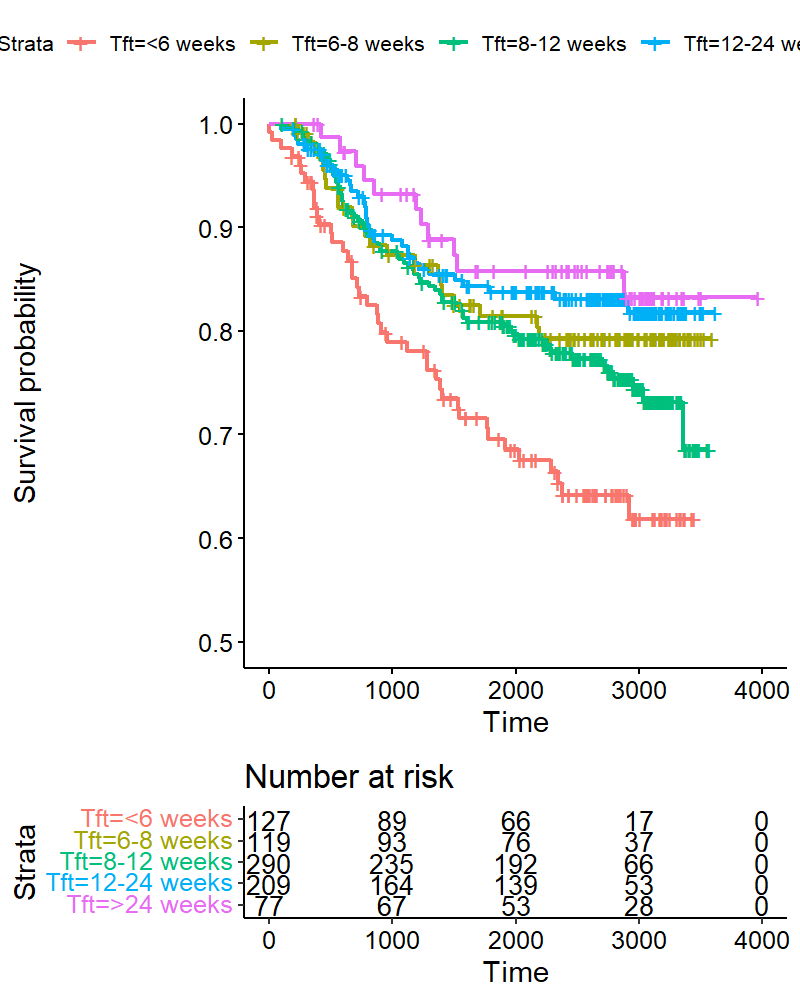

Supplement: oyac050_suppl_Supplementary_Figures [file oyac050_suppl_supplementary_figures.docx]
